# Combination therapy of varenicline with nicotine replacement therapy is better than varenicline alone: a systematic review and meta-analysis of randomized controlled trials

**DOI:** 10.1186/s12889-015-2055-0

**Published:** 2015-07-22

**Authors:** Ping-Hsun Chang, Chien-Hsieh Chiang, Wei-Che Ho, Pei-Zu Wu, Jaw-Shiun Tsai, Fei-Ran Guo

**Affiliations:** Department of Family Medicine, National Taiwan University Hospital, 7 Chung-Shan South Road, Taipei, 10016 Taiwan; Department of Family Medicine, National Taiwan University Hospital and College of Medicine, 7 Chung-Shan South Road, Taipei, 10016 Taiwan; Department of Community and Family Medicine, National Taiwan University Hospital Yun-Lin Branch, 579, Sec. 2, Yunlin Road, Yunlin, 640 Taiwan; Department of Community and Family Medicine, National Taiwan University Hospital Hsin-Chu Branch, 25, Lane 442, Sec. 1, Jingguo Rd., Hsinchu, 30059 Taiwan

**Keywords:** Smoking cessation, Combination therapy, Varenicline, Nicotine replacement therapy, Systematic review, Meta-analysis

## Abstract

**Background:**

Smoking is a major preventable cause of morbidity and premature death worldwide. Both varenicline and nicotine replacement therapy (NRT) help achieve smoking cessation. However, limited evidence exists regarding whether combination of varenicline and NRT is more effective than either alone. The aim of this research was to investigate the efficacy and safety of varenicline combined with NRT.

**Methods:**

A systematic search of MEDLINE, EMBASE, ClinicalTrial.gov, and Cochrane Library was conducted in November 2014. Two authors independently reviewed and selected randomized controlled trials. The quality of the studies was evaluated by the Jadad score. We carried out meta-analysis of both early (abstinence rate assessed before or at the end of treatment) and late (assessed after the end of the treatment) outcomes.

**Results:**

Three randomized controlled trials with 904 participants were included in this meta-analysis. All three were comparing combination therapy with varenicline therapy alone. The late outcomes were assessed in 2 of the 3 trials. Both the early and late outcomes were favorable for combination therapy (OR = 1.50, 95 % CI 1.14 to 1.97; OR = 1.62, 95 % CI 1.18 to 2.23, respectively). However, this significance diminished after eliminating a study with pre-cessation treatment using nicotine patch. The most common adverse events were nausea, insomnia, abnormal dreams, and headache. One study reported more skin reactions (14.4 % vs 7.8 %; *p* = 0.03) associated with combination therapy.

**Conclusions:**

Combination therapy is more effective than varenicline alone, especially if pre-cessation treatment of nicotine patch is administrated. Adverse events of combination therapy are similar to mono-therapy except for skin reactions.

## Background

Smoking is a leading preventable cause of morbidity and premature death worldwide [1]. It has been well established that smoking increases risk of respiratory disease, cardiovascular disease, diabetes mellitus, autoimmune disorders, reproductive system disorders, and many kinds of cancers [2]. Varenicline not only acts as a partial agonist to attenuate withdrawal symptoms during smoking cessation, but also as an agent to block nicotine binding [3]. In a guideline proposed to treat tobacco use and dependence in 2008, seven first-line medications were recommended (nicotine in the forms of gum, inhaler, lozenge, nasal spray and patch, sustained release bupropion hydrochloride, and varenicline) [4]. Among them, varenicline had the highest abstinence rate. A meta-analysis revealed that varenicline was more effective than standard-dose nicotine replacement therapy (NRT) (relative risk = 1.38, 95 % CI 1.15 to 1.64 at 6 months), but was similar to high-dose NRT (relative risk = 1.05, 95 % CI 0.80 to 1.36 at 6 months) [5]. In another meta-analysis, although varenicline was still regarded as the most effective mono-therapy, it was not superior to combination therapy of two different types of NRT [odds ratio (OR) = 1.06, 95 % CI 0.75 to 1.48] [6].

Combination therapy of varenicline with other medications was not recommended in the guideline proposed by the National Institute for Health and Care Excellence (NICE) [7]. Combination therapy of varenicline with NRT is not recommended either by the US Public Health Clinical Practice Guideline for Treating Tobacco Use and Dependence [4]. The efficacy of combination therapy was inconsistent. A retrospective study revealed that the combination therapy of varenicline and NRT was tolerable, but was not superior to mono-therapy [8]. One randomized controlled trial (RCT) showed that varenicline combined with nicotine patch was more effective than varenicline alone to achieve continuous abstinence rate at 12 and 24 weeks [9]. However, another two RCTs showed no superior effects [10, 11]. This research aimed to evaluate the efficacy and safety of varenicline combined with NRT through a systematic review and meta-analysis of RCTs.

## Methods

### Search strategy

We conducted a comprehensive search in November 2014. The databases included MEDLINE (from 1966 to November 2014), EMBASE (from 1966 to November 2014), ClinicalTrail.gov (from 2000 to November 2014) and the Cochrane Library. Search terms were varenicline, nicotine replacement therapy (including nicotine patch, gum, inhaler, nasal spray, lozenge). We combined “varenicline” and “nicotine replacement therapy” by the Boolean operator “and” for screening in titles, abstracts and key words. Then the results were limited to “randomized controlled trial”. After selecting articles, we searched the reference lists for relevant citations. We limited the language to English. We did not limit countries of publications.

### Selection criteria

Only published RCTs with an adult population were included. Trials had to investigate combination treatment of varenicline and nicotine replacement therapy. The required outcomes were abstinence rates with biochemical verification, safety profile, or tolerability of the therapy. Exclusion criteria included non-RCT studies, trials without outcome measurements, trials using smoking cessation medications but not aiming to stop cigarette smoking (eg. stop alcohol use or long term NRT use), and articles that the full-text was not available.

### Study selection

One author (WC) searched the electronic databases. The results were independently assessed by two authors (WC and PZ). The authors identified articles eligible for further review by screening the titles and abstracts. The second step of selection was based on the full-text of articles. The disagreements were resolved by consensus between authors. A standardized data extraction form was used to collect population characteristics, study inclusion and exclusion criteria, intervention details, and outcome data from each study.

### Data extraction

Information was extracted from each included trial on: (1) characteristics of trial participants (including age, sex, location, Fagerström Test for Nicotine Dependence (FTND) score), and the trial’s inclusion and exclusion criteria; (2) type of intervention (including type, dose, duration and frequency of varenicline and NRT; behavioral counseling); (3) type of outcome measure (exhaled carbon monoxide, self-reporting), length of follow-up, adverse effects. One author (WC) extracted the data from included studies and the second author (PZ) checked the extracted data. Disagreements were resolved by discussion between the two review authors; if no agreement could be reached, it was planned a third author (PH) would decide.

### Quality assessment and publication bias

The quality of the studies was assessed by the Jadad score [12]. The score ranges from 0 to 5 according to randomization, blinding, and patient dropout. We assessed the risk of publication bias by funnel plots. Asymmetry in a funnel plot was considered as a risk of publication bias. We used Begg’s rank correlation test and Egger’s regression test for statistical verification of bias [13]. The test results were generated by Comprehensive Meta-Analysis Version 2 software (Biostat, Englewood, NJ, USA).

### Statistical analysis

Pooled ORs were used to compare the effects of treatments. We defined the early outcome as the quit rate assessed before or at the end of treatment completion. The late outcome was the quit rate assessed for a period of time after the end of treatment completion, majorly at 24 or more weeks. The case numbers of adverse events were aggregated and the event rates were expressed as percentages. We calculated the ORs and 95 % confidence intervals (95 % CI) of adverse events by fixed effect model. Between-study heterogeneity was estimated using the χ^2^-based Q statistic [14] and heterogeneity was considered statistically significant when P-value was less than 0.1. If heterogeneity was significant, the pooled estimate was calculated based on the random effects model [15]. In the absence of significant heterogeneity, the pooled estimate was calculated using the fixed effect model [16]. A statistical test with a *P* value less than 0.05 was considered significant in pooled estimates. The forest plots and pooled estimates were generated by Review Manager (RevMan Version 5.3 Copenhagen: The Nordic Cochrane Centre, The Cochrane Collaboration, 2014).

## Results

### Study identification

**Fig. 1:**
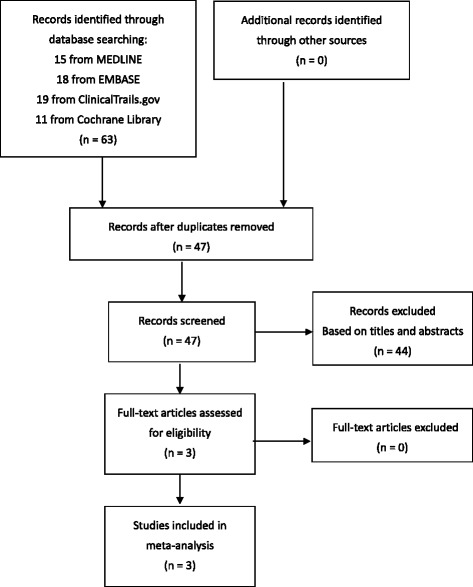
Flow diagram of meta-analysis: inclusion and exclusion of studies

The search strategy retrieved a total of 63 citations. Of these, three were appropriate for full-text review (Fig. 1). No additional study was identified after searching the reference lists of these three articles. After review, all three studies [9–11] were finally included in the systematic review and meta-analysis (Table 1). These three studies were all combination therapy versus varenicline alone therapy trials.

**Table 1:** Baseline characteristics of the included studies

Source	Participants	Mean age	Male %	FTND^a^	Location	Jadad score	Intervention	Early outcome	Late outcome
Hajek [10]	Active: 58	44.5	66.7 %	4.9	London, UK, 1 center	4	15 mg/16 hours nicotine patch, started on TQD, continued to 4th week	1-4 week continuous abstinence	Not available
	Placebo: 59						Varenicline 1 week before TQD, titrated to 2 mg/day, continued to 12th week		
	Total: 117								
Koegelenberg [9]	Active: 216	46.3	38.3 %	4.5	South Africa, 7 centers	5	15 mg/16 hours nicotine patch, started 2 weeks before TQD, continued to 12th week.	9-12 week continuous abstinence	9-24 week continuous abstinence
	Placebo:219						Varenicline 1 week before TQD, titrated to 2 mg/day, continued to 12th week, tapered on the 13th week		
	Total: 435								
Ramon [11]	Active: 170	45.2	57.8 %	6.5	Barcelona, Spain, 1 center	5	21 mg/24 hours nicotine patch, started on TQD, continued to 12th week	2-12 week continuous abstinence	2-24 week continuous abstinence
	Placebo:171						Varenicline 1 week before TQD, titrated to 2 mg/day, continued to 12th week		
	Total: 341								

FTND, Fagerström Test for Nicotine Dependence; TQD, target quit date

^a^ranging from 0 to 10, a higher score denotes greater dependence

### Characteristics of included studies

Table 1 summarizes the baseline demographics, number of participants, interventions, and outcome measurements of included studies. All three trials recruited smokers who were aged 18 and over, not breastfeeding or pregnant, and had no current psychiatric or other serious illness. The inclusion and exclusion criteria were well described in two studies [9, 11], requiring that the enrolled patients have no recent experience of other cessation medication or successful abstinence. The other study provided relatively simple criteria that volunteers seeking treatment with no contraindications could be enrolled [10]. The mean age of participants was similar among all studies. One study included more female subjects than males [9]. There were 38.3 % males in this study vs 66.7 and 57.8 % in the other two studies respectively. The Fagerström Test for Nicotine Dependence (FTND) score was higher in one study because it included smokers who smoked 20 or more cigarettes per day [11], whereas the other two studies included participants with lower daily cigarettes consumption. Treatment interventions differed among these studies. One study administered trial patch two weeks before the TQD, while the other two studies started patch use on the TQD. Two studies used a 15 mg/16 hours patch, while the other [11] used a 21 mg/24 hours patch. On the other hand, the use of varenicline was similar among the studies. All started at 0.5 mg per day one week before TQD, with increase to 2 mg/day on TQD, and continued for 12 weeks. One study [9] tapered the dose of varenicline on the 13th week. All studies provided concurrent behavioral counseling during the treatment phase. One study [10] measured the early outcome at 4 weeks; the other two measured it at 12 weeks. All studies used exhaled carbon monoxide to confirm continuous abstinence. For the late outcome, one study [9] measured 9 to 24 weeks of continuous abstinence, one [11] measured 2 to 24 weeks of continuous abstinence, and the other one did not measure outcome after treatment phase. One study [10] measured self-reported point prevalence at 12 weeks, which was not included in our meta-analysis. All studies adopted our definition of early outcome as the primary outcome, while our defined late outcome as the secondary outcome.

### Study quality and publication bias

The Jadad score of included studies ranged from 4 to 5. Only one study was granted a score of 4 because the dropout of patients was not detailed in the article [10]. The overall quality of the included studies was high. The funnel plot of early outcome was not symmetrical due to lack of smaller studies with positive effect. However, the power of the funnel plot was compromised by the small number of studies. The publication bias assessed by Begg’s rank correlation test and Egger’s regression test was not significant (*p* = 0.30 and 0.28 respectively). The publication bias of late outcome was not assessed because there were only two studies included. The Comprehensive Meta-Analysis software could not generate a funnel plot or a test report under this situation.

### Varenicline plus nicotine patch versus varenicline plus placebo patch: the early outcome

**Fig. 2:**
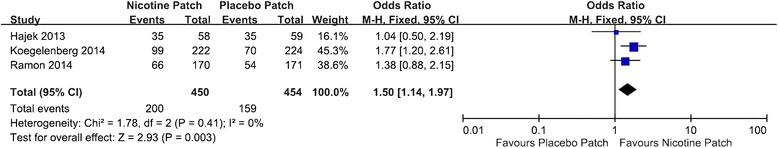
Varenicline plus nicotine patch vs varenicline plus placebo patch: the early outcome

Three studies with a total of 904 participants were included in this meta-analysis (Fig. 2). The heterogeneity was minimal (I^2^ = 0 %, *p* = 0.41), therefore fixed effect model was used. The results demonstrated a significant increase in abstinence rate (44.4 % vs 35.1 %, OR = 1.50, 95 % CI 1.14 to 1.97). All three studies showed a favorable effect of combination therapy, while only one of them reached statistical significance [9].

### Varenicline plus nicotine patch versus varenicline plus placebo patch: the late outcome

**Fig. 3:**
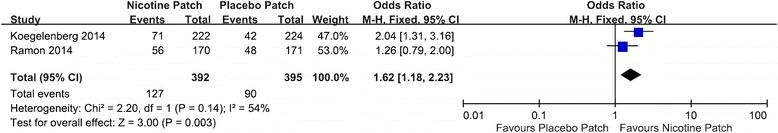
Varenicline plus nicotine patch vs varenicline plus placebo patch: the late outcome

Two studies with a total of 787 participants were included in this meta-analysis (Fig. 3). The heterogeneity was moderate (I^2^ = 54 %) without statistical significance (*p* = 0.14). The fixed effect model was also used. The results demonstrated a significant increase in abstinence rate (32.4 % vs 23.1 %, OR = 1.62, 95 % CI 1.18 to 2.23). Both studies showed a favorable effect of combination therapy, while only one of them reached statistical significance [9].

### The safety of combination therapy

The case numbers of adverse events were aggregated. The pooled ORs were generated by fixed effect model (Table 2). Combination therapy reported more nausea (28.4 % vs 25.7 %), insomnia (18.7 % vs 15.4 %), abnormal dreams (13.6 % vs 10.7 %), but less headache (7.1 % vs 7.8 %). There were no significant differences between nicotine and placebo patch groups. Only one study reported the adverse events of depression, especially in the nicotine patch group (2.3 % vs 1.4 %; *p* = 0.50) [9]. This study also reported more skin reactions in the nicotine patch group (14.4 % vs 7.8 %; *p* = 0.03).

**Table 2:** Adverse events reported in the included studies

	Varenicline + nicotine patch n (%)^a^	Varenicline + placebo patch n (%)^a^	OR (95 % CI)^a^
Nausea	123 (28.4)	113 (25.7)	1.15 (0.85, 1.56)
Insomnia	83 (18.7)	69 (15.4)	1.27 (0.89, 1.80)
Abnormal dreams	51 (13.6)	44 (10.7)	1.20 (0.78, 1.84)
Headache	30 (7.1)	30 (7.8)	1.01 (0.60, 1.72)

^a^Pooled event rates and odds ratios calculated by fixed effect model

A total of eight serious adverse events (SAEs) were reported in the included studies, where only one of them was considered relevant to the study medications. This was a female participant who became pregnant during the treatment phase where she was randomized to receive placebo patch combined with varenicline. She later on gave birth to an infant with Down syndrome and congenital heart defects. Another SAE was a female participant who also became pregnant during treatment phase with placebo patch. She had an anembryonic pregnancy and this SAE was considered to be irrelevant to the study medications.

### Subgroup and sensitivity analysis

We did not perform subgroup analysis because of the small number of studies, and there was no significant heterogeneity. One RCT was identified to be different in study design (pre-cessation treatment with patch) and participant characteristics (more females than males) [9]. When we eliminated this RCT from the meta-analysis model of the early outcome, the favorable effect of combination therapy became insignificant (OR = 1.28, 95 % CI 0.87 to 1.87). We then preformed sensitivity analysis of the late outcome in the same manner and the favorable effect of combination therapy also diminished (OR = 1.26, 95 % CI 0.79 to 2.00). We compared the results of fixed effect to random effects model of both early and late outcomes. The OR was 1.50 (95 % CI 1.14 to 1.97) vs 1.50 (95 % CI 1.14 to 1.97) in the early outcomes, 1.62 (95 % CI 1.18 to 2.23) vs 1.61 (95 % CI 1.00 to 2.58) in the late outcomes respectively. The conclusion of favorable effect persisted even using different models.

## Discussions

Our research identified three smoking cessation trials comparing varenicline combined with nicotine patch versus varenicline combined with placebo patch. No other types of nicotine product were combined with varenicline in these trials. We found no trials comparing combination therapy with NRT alone that met our inclusion and exclusion criteria. One RCT that compared combination therapy and NRT was not included because it combined varenicline and counseling to get long-term NRT users to quit NRT [17]. After meta-analysis, our results demonstrated favorable effects of combination therapy in both early and late outcomes. To our knowledge, this is the first systematic review and meta-analysis on this issue.

In our main analysis, the early outcomes in varenicline combined with placebo patch group were consistent with previous studies using varenicline mono-therapy (abstinence rate 44.4 % vs 43.9 %) [18]. The late outcomes were also similar (abstinence rate 32.4 % vs 29.7 %). In sensitivity analysis, we identified the largest RCT [9] which markedly influenced the results. After eliminating this RCT, the favorable effects of both early and late outcomes became insignificant. We identified two distinguishing factors that might have caused this difference. First, there were more female subjects in this RCT. Previous studies have revealed that the effect of varenicline did not differ among genders [19–21]. On the other hand, some studies yielded inconsistent reports on whether male subjects using NRT had higher abstinence rates than females [22–25]. If nicotine patch use had been less effective in females, the higher percentage of females in this RCT would bring about less effective outcomes, which was not the case. Therefore, gender difference did not seem to contribute to the treatment effect of this RCT.

Second, this RCT [9] used a pre-cessation nicotine patch. A meta-analysis conducted by Shiffman S *et al.* showed that pre-cessation nicotine patch significantly increased abstinence rates at 6 weeks (OR = 1.96, 95 % CI 1.31 to 2.93) and at 6 months (OR = 2.17, 95 % CI 1.46 to 3.22) [26]. However, this positive effect was not consistent in the following meta-analyses [6, 27, 28]. These meta-analyses showed that pre-cessation nicotine patch and gum had a moderate but insignificant increase in abstinence rates. Pre-cessation nicotine patch appeared to be more effective than pre-cessation nicotine gum [27]. Therefore, we favored that the effect of pre-cessation nicotine patch contributed to the better outcomes in this RCT.

The rationale of combination therapy of varenicline with NRT resides in the hypotheses that 1) varenicline does not fully saturate α4β2 nicotinic acetylcholine receptors; 2) varenicline does not completely replace the dopaminergic effect of smoking [29]. A standard-dose of varenicline (1.0 mg) could achieve higher abstinence rates than low-dose varenicline (0.5 mg) [3]. Further saturation of the receptors seemed to explain the additive effect of NRT. However, a neuropharmacological study utilizing positron emission tomography revealed that a single dose of 0.5 mg varenicline could saturate α4β2 receptors in the human brain [30]. It deserves a debate whether the combination to α4β2 nicotinic acetylcholine receptors, and subsequent mesolimbic dopamine release is the only pathway that causes reward in smoking. Nicotine addiction develops from complex pathways, and individual genotypes influence both smoking behavior and treatment effects [31, 32]. The modulation of α4β2 receptors might not be the only pathway to release dopamine, and dopamine might not be the only neurotransmitter involved in smoking behavior. More studies are required to explore the mechanism of combining varenicline with NRT.

The event rates of adverse effects were similar in the two groups. The only significant increase in adverse events was skin reactions in one RCT [9]. The event rate of skin reactions was comparable to those in nicotine patch mono-therapy [28]. Other adverse events of combination therapy were not higher than findings from previous studies of varenicline mono-therapy [3]. The birth of an infant with Down syndrome (trisomy 21) in one study [9] was considered relevant to varenicline, classified as a Pregnancy Category C drug. Koegelenberg *et al.* considered that the causality was less likely because the association was not observed in post-marketing researches [33, 34]. Varenicline combined with nicotine patch appeared to be safe and tolerable in smoking cessation.

### The question of small study number

Critics might argue that it was too early to perform a meta-analysis when there were only three RCTs available. Borenstein *et al.* suggested that it makes sense to perform a meta-analysis as soon as we have two studies, since a summary based on two studies yields a more precise estimate of the true effect than either study alone [35]. However, the estimate of the pooled effects would have poor precision. Three solutions are suggested under such circumstance [36]. One option is to report the separate effects and not report a summary effect. Readers are expected to understand that authors cannot draw conclusions about the effect size. The problem is that some readers may revert to ‘vote counting’ and possibly reach an erroneous conclusion. Another option is to perform a fixed effect analysis. This approach yields a descriptive analysis of the included studies, but does not allow us to make inferences about a wider population. A third option is to take a Bayesian approach, where the estimate of variance is based on data from outside of the current studies. We selected the second approach and used fixed effect model in our analysis.

### Strengths and limitations

In our study, we searched the major databases with rigorous strategies. There were duplicated authors who selected the articles independently, allowing for a low probability that an important study was missed. The included studies had high quality and the Jadad score ranged from 4 to 5. The heterogeneity between the selected studies was low, and there were no significant publication biases. We evaluated both early and late outcomes which was more comprehensive than short-term evaluations. However, there were some limitations. We did not search grey literature or un-published data. Trials that were less known might have been missed. The strength of our research was compromised by the small number of trials. The largest RCT which had the greatest influence to our results was different from the other RCTs in demographic characteristics and treatment design. The impact was that our conclusions could not be generalized to other populations. Also, the funnel plot and tests of publication bias had low power to detect a potential bias. In our review, the adverse events of depression and skin reactions were only reported in one study. There was no report of cardiovascular or suicidal events. The safety of combination therapy requires further investigations.

## Conclusions

The combination therapy of varenicline with NRT is more effective than varenicline alone in smoking cessation. This effect is more evident if pre-cessation treatment of nicotine patch is administrated. The adverse events of combination therapy are comparable to varenicline mono-therapy with the exception of skin reactions. Larger RCTs are needed to make more robust conclusions.

## Abbreviations

NRT: Nicotine replacement therapies
RCT: Randomized controlled trial
OR: Odds ratio
95 % CI: 95 % confidence interval
FTND: Fagerström Test for Nicotine Dependence
TQD: Target quit date
